# High prevalence of lack of knowledge and unhealthy lifestyle practices regarding premature coronary artery disease and its risk factors among the Saudi population

**DOI:** 10.1186/s12889-023-15834-1

**Published:** 2023-05-19

**Authors:** Thamir Al-khlaiwi, Hessah Alshammari, Syed Shahid Habib, Razan Alobaid, Lama Alrumaih, Alaa Almojel, Faye Sendi, Shahad Almuqbil, Majdoly Alkhodair

**Affiliations:** 1 Department of Physiology, College of Medicine, King Saud University, Riyadh, Kingdom of Saudi Arabia; 2 Department of Cardiology, College of Medicine, King Saud University, Riyadh, Kingdom of Saudi Arabia; 3 Medical student, College of Medicine, King Saud University, Riyadh, Kingdom of Saudi Arabia

**Keywords:** Premature coronary artery disease (PCAD), Knowledge, Attitude, Practice

## Abstract

**Objective:**

Literature regarding coronary artery disease (CAD) and awareness of its risk factors is available in Saudi Arabia (SA). However, it is lacking with respect to premature coronary artery disease (PCAD). Therefore, it is necessary to evaluate the lack of knowledge of this underrepresented critical issue and to devise a well-constructed strategy for PCAD. This study aimed to assess the knowledge of PCAD and its risk factors in SA.

**Methods:**

A cross-sectional questionnaire-based study was performed in the Department of Physiology, College of Medicine, King Saud University (KSU), Riyadh, SA between July 01, 2022, and October 25, 2022. A validated proforma was sent to the Saudi population. The sample size was 1046 participants.

**Results:**

Proforma results indicated that 46.1% (*n* = 484) of participants believed that CAD could occur in people under the age of 45, whereas 18.6% (*n* = 196) did not believe, and 34.8% (*n* = 366) did not know. There was a highly statistically significant association present between sex and the belief that CAD can affect people who are under the age of 45 (*p* < 0.001), with 355 (73.3%) females believing that CAD can affect people below the age of 45 compared to 129 (26.7%) males. The results also showed a highly statistically significant relationship between educational status and the belief that CAD can affect people who are under the age of 45 (bachelor’s degree, 392 participants, representing 81.1%; *p* < 0.001). Furthermore, having employment was notably positively associated with that belief (*p* = 0.049) as was having a health specialty (*p* < 0.001). In addition, 62.3% (*n* = 655) of participants were not aware of their lipid profile, 49.1% (*n* = 516) preferred using vehicles to get to nearby places, 70.1% (*n* = 737) did not undergo regular medical checkups, 36.3% (*n* = 382) took medications without consultations, 55.9% (*n* = 588) did not exercise weekly, 69.5% (*n* = 112) were E-cigarette smokers, and 77.5% (*n* = 810) consumed fast food weekly.

**Conclusions:**

Individuals from SA have an evident lack of public knowledge and poor lifestyle practices regarding PCAD, which demonstrates the need for health authorities to take a more targeted and attentive approach toward PCAD awareness. In addition, extensive media involvement is required to highlight the severity of PCAD and its risk factors in the population.

## Introduction

Cardiovascular diseases (CVDs) are the leading cause of death throughout the world, accounting for 32% of all deaths worldwide, with an estimated 17.9 million lives each year [1, 2]. CVDs are also becoming an increased health concern in Gulf countries, including Saudi Arabia (SA) [3]. In SA, chronic diseases result in 78.0% of all deaths [4, 5]. Among these deaths, CVDs amount to 45.7% [4, 5]. CAD is the most prevailing type of heart disease and continues to be the leading cause of death worldwide, accounting for more than 350,000 deaths annually [2, 6]. In 2021, an American Heart Association (AHA) report showed that the total CAD prevalence is 7.2% in US adults ≥ 20 years of age, with a male prevalence of 8.3% and a female prevalence of 6.2% [7]. In a study from SA, it was reported that the total prevalence of CAD is 5.5%, with a prevalence in males and females of 6.6% and 4.4%, respectively [8]. A major complication of CAD is myocardial infarction (MI), which can present as sudden death. MI mostly occurs in patients older than 45; however, MI can also affect the younger population [9]. In fact, 4–10% of people with CAD are younger than 45 years, which is known as PCAD [9]. In most studies, the age of 45 has been used as the cut off to define patients with PCAD [10].

A recent study conducted in SA revealed that 49% of the total cases of ST-elevation myocardial infarction (STEMI) involved patients under 45 years of age [11]. Another cross-sectional study compared the prevalence of cardiovascular risk factors among Saudi and Egyptian medical students between the ages 18–25 and found that 23.9% of Saudi students and 16.7% of Egyptian students were at high risk of developing CVD within a decade [12]. As medical students are expected to be knowledgeable, these results lead us to believe that there might be deficiencies in knowledge and prevalence of unhealthy lifestyles among health professionals. If this is the case for health professionals, other specialties might have a more evident lack of knowledge and adoption of unhealthy attitudes and practices that need to be assessed.

There are several risk factors that contribute to the development of PCAD; some are traditional risk factors, while others are nontraditional risk factors attributable to genetic predisposition. A recent study conducted in the US found that the most common cardiovascular factor was past or current smoking (60.8%), followed by hypertension (HTN) (52.8%) and family history of CAD (39.8%) [13]. Other studies in an Australian systematic review and meta-analysis compared patients with CAD to those with PCAD and found opium use, family history of CAD, smoking, and dyslipidemia to be prevailing in PCAD, whereas HTN, obesity, diabetes mellitus (DM) and sedentary lifestyle were shown to be more prevalent among those who have CAD [6].

Some studies in China have suggested that genetic predisposition is the main factor in the pathogenesis of PCAD. Ethnicity and race may be involved in inherited variants that lead individuals to develop PCAD [14]. The effects of genetic predisposition are exacerbated by traditional cardiovascular risk factors, such as smoking, DM, HTN, obesity, and dyslipidemia. Accordingly, PCAD patients have a higher prevalence of HTN, higher blood glucose levels, and a higher body mass index (BMI) than healthy individuals [14]. Another systematic review and meta-analysis conducted in Iran showed that DM is the most prevalent and significant risk factor for PCAD among the Iranian population, followed by dyslipidemia and family history of CAD, smoking and HTN. The highest BMI values were observed in patients with PCAD [15].

A recent study was conducted in 2020 on 311 subjects and showed that most of the participants displayed a lack of knowledge about CAD (82%) and were unfamiliar with any risk factors (26.4%), symptoms (25.1%), and complications (72.7%) of CAD [16]. However, the study population was restricted to Saudi residents who live in Dawadmi city and the villages around it. A lack of awareness about risk factors for the disease was observed in a study conducted in India in 2021 that evaluated the risk factors for PCAD [17]. Although the participants were aware of the disease itself, their lack of awareness of its risk factors raises concern.

Despite our extensive efforts, we have not found studies that thoroughly investigated the awareness of PCAD in SA despite its serious consequences and the possibility that it can be prevented.

A study to assess knowledge, attitudes, and practices regarding PCAD risks is crucial due to the modifiable nature and devastating consequences of PCAD. Therefore, this study aimed to assess the knowledge of PCAD and its risk factors in SA.

## Methods

The study was performed in the Department of Physiology, College of Medicine, KSU, Riyadh, SA between July 01, 2022, and October 25, 2022. The study design was an observational, cross-sectional questionnaire-based study conducted through a random sampling technique among the Saudi population. The questionnaire was translated from English to Arabic by the authors, and the appropriateness of the translation was tested through a pilot study. It was also reviewed by a native cardiologist. The pilot study was conducted with 20 proforma relying on the author networks that were sent through WhatsApp, and it was also sent to a cardiologist. Adjustments were made according to their feedback. Then, the Google questionnaire was distributed randomly through social media and in person. The social media platforms used were WhatsApp, Twitter, and Telegram. The authors also collected data in-person through visiting universities and malls. The universities were King Saud, Princess Noura, Imam Mohammad Ibn Saud Islamic, Alfaisal, and Prince Sultan. The colleges visited were Humanity, Business, Health, Science, Engineering, and Technical. Data were collected during the unified lunch break (12:00 pm-1:00 pm) from available subjects who agreed to participate. Hayat Mall, Panorama Mall and Nakheel Mall (Riyadh, SA) were also included. We approached people in the food court who agreed to participate. This was done over a duration of 2 months. Participation was voluntary and anonymous, and consent to participation was obtained by filling in the proforma. The inclusion criteria were Saudi citizens living in SA aged 18–45 years. Exclusion criteria were being non-Saudi or living outside SA. This choice was made for the accurate measurement of outcomes, as there are ethnic differences among risk factors for PCAD that can distort the results [11].

The sample size was calculated to be 1000 participants. We distributed more than 2500 questionnaires, and 1046 participants responded, and these were eligible to be included according to the inclusion criteria. The study adhered to the tenants of the Declaration of Helsinki. In addition, the study was approved by the ethics committee of the KSU (approval on Research Project No. E-22-6747).

Overall, four themes were included in the questionnaire: personal information, knowledge of PCAD, attitude, and practice. Personal information contained sociodemographic characteristics such as age, sex, educational status, occupation, BMI, marital status, and specialty. Knowledge of PCAD was measured by a direct question. Additionally, questions regarding knowledge of the risk factors for PCAD, attitudes of the participants, and practices to address those issues were included. A 3-point Likert scale (Yes, no, do not know) was used.

The study was approved by The Institutional Review Board (IRB), College of Medicine Research Centre (CMRC), KSU, Riyadh, SA (E-22-7064).

### Data analysis

Analysis was performed with SPSS 25 version statistical software. Categorical and quantitative variables were described using frequencies and percentages. We used Pearson’s chi-square test to evaluate the correlation between categorical and outcome variables. A *p* value of ≤ 0.05 and 95% confidence intervals were calculated to determine statistical significance.

## Results

In this study, 1046 validated proforma were retrieved for analysis. The sociodemographic data are presented in Table 1, showing that there were 973 participants aged below 29 years, representing 92.5% of the sample. With regard to sex, 401 were male, representing 38.2% of the sample, and 858 (81.6%) had a bachelor’s degree. A total of 212 (20.2%) participants were health professionals. Regarding occupation, 115 (10.9%) of the participants reported being employed. Regarding BMI, 582 participants (55.4%) reported an ideal BMI, while 347 (33.1%) were overweight and obese. Twenty-eight respondents, representing 2.7%, mentioned having a family member or a friend who has CAD and is below the age of 45.

**Table 1:** Sociodemographic characteristics of the sample (*N* = 1046)

Variables	N (%)
Age	
< 20	322 (30.8)
20–29	651 (62.2)
30 and over	73 (7.0)
Sex	
Male	401 (38.3)
Female	645 (61.7)
Marital status	
Married	85 (8.1)
Single	961 (91.9)
University	
Governmental	992 (94.8)
Private	54 (5.2)
Educational status	
High school	78 (7.5)
Bachelor	858 (82)
Postgraduate	110 (10.5)
Occupation	
Employed	115 (11)
Unemployed	931 (89)
Specialty	
Humanity College	207 (19.8)
Business College	187 (17.8)
Health College	212 (20.3)
Sciences College	144 (13.8)
Technical College	147 (14.1)
Engineering College	132 (12.6)
Unspecified	17 (1.6)
BMI	
< 20	117 (11.2)
20-24.9	582 (55.6)
25-29.9	257 (24.6)
30-34.9	64 (6.1)
35-39.9	19 (1.8)
40-45.9	7 (0.7)
Smoking (*n* = 161, 15.3% of the total)	
Mostly traditional smoking	61 (37.9)
Mostly E-cigarette	112 (69.6)
Mostly Shisha	52 (32.3)
Do you have family/friends under the age of 45 that have been diagnosed with coronary artery disease?	
Yes	28 (2.7)
No	1018 (97.3)

Data are represented as numbers and percentages

As shown in Table 2, 46.1% (*n* = 484) of participants thought that CAD can affect people under the age of 45, whereas 18.6% (*n* = 196) did not agree, and 34.8% (*n* = 366) did not know. Moreover, only 356 participants (33.9%) believed that complications that result from CAD affecting people under 45 years are more severe than complications that occur in patients older than 45 years. The participants believed that smoking (72%), lack of exercise (69.5%), obesity (74.5%), HTN (62.5%), high levels of cholesterol (70.3%), hyperlipidemia (71.6%), consumption of fast food, (58.2%), and genetic predisposition to heart diseases (73.3%) can cause CAD in people under 45 years of age. On the other hand, only 397 (37.8%) and 393 (37.4%) participants believed that opioids and DM, respectively, can cause CAD in people under 45 years of age.

**Table 2:** Knowledge of participants toward PCAD and its risk factors, *N* = 1046

Knowledge	Yes n (%)	No n (%)	I do not Know n (%)
Coronary artery disease can affect people who are under the age of 45?	484 (46.1)	196 (18.6)	366 (34.8)
Complications that result from coronary artery disease affecting people under 45 years are more severe than complications that occur in patients older than 45?	356 (33.9)	271 (25.8)	419 (39.9)
Coronary artery disease affecting people under 45 can lead to death?	532 (50.6)	130 (12.4)	384 (36.5)
Coronary artery disease affecting people under 45 years is a major concern in KSA?	387 (36.8)	226 (21.5)	433 (41.2)
Smoking can cause coronary artery disease in people under 45 years?	757 (72.0)	80 (7.6)	209 (19.9)
Lack of exercise can cause coronary artery disease in people under 45 years?	730 (69.5)	124 (11.8)	192 (18.3)
Obesity can cause coronary artery disease in people under 45 years?	783 (74.5)	74 (7.0)	189 (18.0)
Hypertension can cause coronary artery disease in people under 45 years?	657 (62.5)	98 (9.3)	291 (27.7)
Diabetes can cause coronary artery disease in people under 45 years?	393 (37.4)	225 (21.4)	428 (40.7)
High levels of cholesterol can cause coronary artery disease in people under 45 years?	739 (70.3)	56 (5.3)	251 (23.9)
Hyperlipidemia (triglycerides) can cause coronary artery disease in people under 45 years?	753 (71.6)	49 (4.7)	244 (23.2)
Consumption of fast food can cause coronary artery disease in people under 45 years?	612 (58.2)	192 (18.3)	242 (23.0)
Family history (genetic predisposition) of heart diseases can cause coronary artery disease in people under 45 years?	770 (73.3)	61 (5.8)	215 (20.5)
Family history (genetic predisposition) of hypercholesterolemia can cause coronary artery disease in people under 45 years?	655 (62.3)	96 (9.1)	295 (28.1)
Life stressors can increase the risk of coronary artery disease in people under 45 years?	542 (51.6)	231 (22.0)	273 (26.0)
Some medications can cause coronary artery disease in people under 45 years?	389 (37.0)	233 (22.2)	424 (40.3)
Using opioids can cause coronary artery disease in people under 45 years?	397 (37.8)	129 (12.3)	520 (49.5)

Table 3 indicates a statistically significant association between sex and the belief that CAD can occur in people who are under the age of 45 *(p* < 0.001), with 355 (73.3%) females believing that CAD can affect people below the age of 45, compared to 129 (26.7%) males. There was also a positive statistically significant relationship between educational status and the belief that CAD can affect people who are under the age of 45 (bachelor’s degree, 392 participants, representing 81.1%; *p* < 0.001). Furthermore, employed participants showed a significant association with that belief (*p* = 0.049). In addition, participants with a health specialty appeared to have a positive association with that belief (*p* < 0.001).

**Table 3:** Correlation of variables with knowledge of PCAD (Do you believe that coronary artery disease can affect people who are under the age of 45?), *N* = 1046

Parameter	Yes	No	Do not know	*p* value
**Sex**				
Male	129 (26.7%)	162 (82.7%)	110 (30.1%)	< 0.001*
Female	355 (73.3%)	34 (17.3%)	256 (69.9%)	
**Educational status**				
High School	31(6.4%)	5 (2.6%)	42 (11.5%)	< 0.001*
Bachelor	392 (81.1%)	180 (91.8%)	286 (78.2%)	
Postgraduate	61 (12.5%)	11 (5.6%)	38 (10.3%)	
**Specialty**				
Humanity	88 (18.8%)	39 (19.9%)	80 (21.9%)	< 0.001*
Business	66 (13.6%)	24 (12.2%)	97 (26.5%)	
Health	169 (34.9%)	14 (7.1%)	29 (7.9%)	
Sciences	52 (10.7%)	43 (21.9%)	49 (13.4%)	
Technical	50 (10.3%)	36 (18.4%)	61 (16.7%)	
Engineering	51 (10.5%)	40 (20.4%)	41 (11.2%)	
Nonspecific	8(1.7%)	0(0.0%)	9(2.5%)	
**Occupation**				
Employed	43 (8.9%)	30 (15.3%)	42 (11.5%)	0.049*
Unemployed	441 (91.9%)	166 (84.7%)	324 (88.5%)	

Data are represented as number and percentage, Chi-squared test: * Significant at *p* ≤ 0.05

**Fig. 1:**
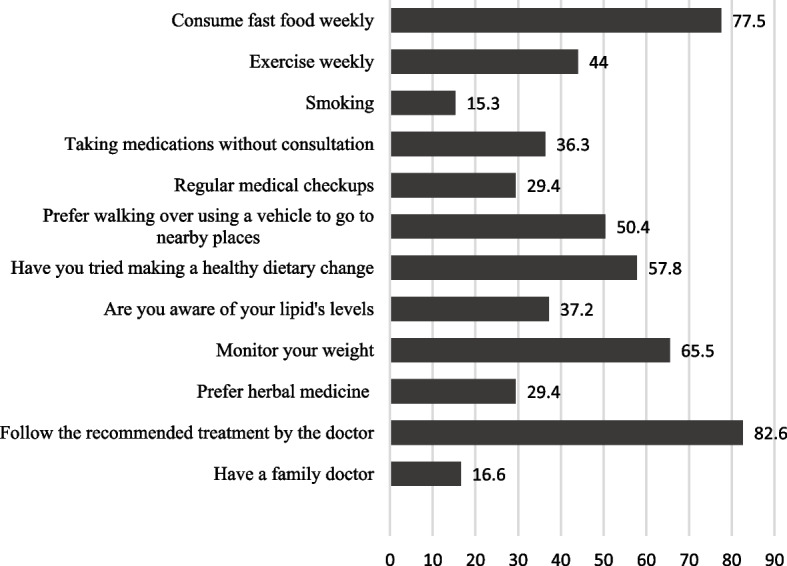
Practice and attitude of participants, *N* = 1046. Numbers are presented as percentages. Figure 1 shows the practice and attitude of the participants as a percentage, 77.5% (*n* = 810) consume fast food weekly, 55.9% (*n* = 588) do not exercise weekly, smoking 69.5% (*n* = 112), 36.3% (*n* = 382) take medications without consultations, 70.1% (*n* = 737) do not undergo regular medical checkups, 49.1% (*n* = 516) are preferring using vehicles to get to nearby places, 57.8% (*n* = 607) tried making a healthy dietary change, 62.3% (*n* = 655) are not aware of their lipid profile, 65.5% (*n* = 688) regularly monitor their weight, 29.4% (*n* = 309) prefer the use of traditional/herbal medicine not prescribed by a licensed physician, 82.6% (*n* = 868) follow the recommended treatment by the doctor, and only 16.6% (*n* = 165) and have a family doctor

Practices and attitudes of the participants were explored, and the different lifestyle habits of the participants were taken into consideration. The results were astonishing: 77.5% (*n* = 810) consumed fast food weekly, 55.9% (*n* = 588) did not exercise weekly, 69.5% (*n* = 112) smoked, 36.3% (*n* = 382) took medications without consultations, 70.1% (*n* = 737) did not undergo regular medical checkups, 49.1% (*n* = 516) preferred using vehicles to get to nearby places, 57.8% (*n* = 607) tried making a healthy dietary change, 62.3% (*n* = 655) were not aware of their lipid profile, 65.5% (*n* = 688) regularly monitored their weight, 29.4% (*n* = 309) preferred the use of traditional/herbal medicine not prescribed by a licensed physician, 82.6% (*n* = 868) followed the recommended treatment prescribed by their doctor, and only 16.6% (*n* = 165) had a family doctor (Fig. 1).

As shown in Table 4, there were statistically significant differences between males and females for all the risk factors for PCAD, with a *p* value of < 0.001. It also shows statistically significant differences between males and females in the attitude toward healthy lifestyle. Females display a more positive lifestyle than males in having a family doctor (*p* < 0.001), weight monitoring (*p* < 0.001), lipid monitoring (p.02), and consuming a healthy diet (*p* < 0.001). In addition, there were statistically significant differences between males and females in the practices of walking, smoking, exercising, and consuming fast food (*p* < 0.001). Females exercised and walked more, smoked less, and consumed less fast food in general.

**Table 4:** Comparison of sex and knowledge, attitudes and practices toward PCAD risk factors

Sex	Knowledge									Attitude				Practice			
	Smoking	Lack of Exercise	Obesity	HTN	DM	Cholesterol	Hyperlipidemia	Fast food	Family history	Family Doctor	Weight monitoring	Lipid Monitoring	Healthy diet	Walking	Smoking	Exercise	Fast food
Male (%)																	
Yes	70.6	77.6	76.3	64.6	37.7	82.5	81.3	62.6	81.3	9.2	58.6	32.9	39.4	42.4	32.7	S: 80.5 M: 8.5 A: 11 *	0: 27.2 1: 18.5 2: 32.4 3: 21.9 *
No	14.2	11.7	11	13.5	28.4	5	3.7	25.7	3.7	90.8	41.4	67.1	60.6	57.6	67.3		
IDK	15.2	10.7	12.7	21.9	33.9	12.5	15	11.7	15								
Female (%)																	
Yes	73.5	65	74	61.7	37.5	63.3	66.2	56	68.8	21.2	70.2	40.2	69.6	55.8	4.7	S: 65 M: 12.4 A: 22.6	0: 19.7 1: 29.3 2: 31.8 3: 19.2
No	3.6	11.9	4.7	6.8	17.2	5.6	5.3	13.8	7.1	78.8	29.8	59.8	30.4	44.2	95.3		
IDK	22.9	23.1	21.4	31.5	45.3	31.2	28.5	30.2	24								
	< 0.001*	< 0.001*	< 0.001*	< 0.001*	< 0.001*	< 0.001*	< 0.001*	< 0.001*	< 0.001*	< 0.001*	< 0.001*	0.02*	< 0.001*	< 0.001*	< 0.001*	< 0.001*	< 0.001*

* Letters represent the following terms: S = Sedentary, M = Moderate, A = Active

*Numbers represent how many times a week: 0 = Rarely 1 = Once a week 2 = Twice a week 3 = More than twice a week

## Discussion

Although modifiable cardiovascular risk factors such as high blood pressure, DM, smoking, physical inactivity, obesity, dyslipidemia and psychological stress are seen in a higher proportion of PCAD patients, a failure to control those factors is often observed [6, 13]. This could be due to a lack of awareness and knowledge of PCAD and its risk factors. As a matter of fact, almost all CVD outcomes can be prevented through healthy food and lifestyles such as diets low in saturated fat and high in fruits and vegetables as well as exercise and smoking cessation [16]. Literature regarding CAD and awareness of its risk factors is available in SA; however, it is lacking with respect to PCAD.

A lack in individuals’ awareness of the 14 risk factors for CAD was found in Jeddah, Saudi Arabia [18]. Smoking, obesity, hyperlipidemia, and intake of junk food were the most recognized risk factors [19]. In south Dublin, individuals aged 20–30 years showed recognition of CAD, which represented the majority of their study sample. This strikingly contrasts with our findings, as more than half of our participants did not recognize PCAD [20]. In 2014, a review on CAD risk factors in college students found that most young adults were unaware of their risk of developing CAD [21].

Our findings highlight the low knowledge of PCAD in SA, although the majority have a bachelor’s degree, and some are health professionals. Our results were found to be higher than what has been reported in Dawadmi, but their study was only conducted in the city of Dawadmi (a smaller city in Saudi Arabia) and included a small sample size and a focus on CAD [16]. Regarding risk factors, our findings revealed higher awareness and knowledge than in a PCAD study conducted in India [17] and lower awareness and knowledge than what Albadrani et al. found on CAD [19].

We also found a positive correlation between sex and knowledge of PCAD. This finding could be due to the nature of men and their beliefs that during adolescence, people enjoy full health and cannot be affected by cardiac diseases, while females are generally more aware of health concerns due to reported self-consciousness [22]. Whether this is due to the busier schedules, or the nature of men should be properly investigated to understand their poor health attitudes and practices.

More than 30% of the participants believed that complications that result from coronary artery disease affecting people under 45 years are more severe than complications that occur in patients older than 45 years. People who are affected by premature coronary artery diseases are more prone to have more impact on their financial and social situations, while those who are affected by CAD in their later years of life have already established social and financial lives. In addition, the health care burden on governments and on other family members will be higher on PCAD patients because of their younger ages.

Being employed showed a significant association with knowledge of PCAD. In the past few years, inflation has increased tremendously along with monthly expenses, which has created stress on many families, especially during and after COVID-19. Employed people usually have higher education and, most likely, more knowledge about current illnesses. This might necessitate the need to improve awareness about PCAD in the general population, especially among those who have lower education levels.

Awareness among those with bachelor’s degrees was higher than that among postgraduates. This is alarming since postgraduates are on key posts and have more responsibility and busier schedules, so they generally do not concentrate on general health issues. The low knowledge of PCAD among health-related occupations was expected to some extent. The reason behind this is the lack of emphasis on PCAD in the undergraduate curriculum of our local medical colleges.

The most astonishing findings among the practices of the individuals in our sample were that most of the participants were not aware of their lipid profile, did not undergo regular medical checkups or exercise weekly, consumed fast food weekly, and did not have a family doctor. A deeply concerning result is that most smokers are E-cigarette users. It is imperative to implement programs as an educational strategy to correct any misconceptions about the use of E-cigarettes in particular and greatly limit the devastating consequences of the spread of misinformation surrounding this method.

### Strengths and weaknesses

This is the first study in SA to assess the knowledge and practice of PCAD among the Saudi population. Our sample size was large enough, and it reflects the target population, although we expected more respondents. However, one of the weaknesses of the study is that > 92% of the respondents were below the age of 29, which does not precisely reflect other age intervals. In addition, it is a cross-sectional study.

## Conclusions and recommendations

There is an evident lack of public knowledge and prevalent poor lifestyle practices toward PCAD in SA. This attests to the need for more targeted attention from health authorities to increase awareness of PCAD and its risk factors. In addition, unhealthy practices, especially those related to fast food consumption, are prevalent. Extensive media involvement is required to highlight the severity and risk factors for PCAD. As the Dutch philosopher Desiderius Erasmus said, “prevention is better than cure”. Additionally, more emphasis should be placed on PCAD in undergraduate medical programs to prepare health professionals for their careers.

## Data Availability

All data and materials used and analyzed during the current study are available from the corresponding author on reasonable request.
